# RepACNet: A Lightweight Reparameterized Asymmetric Convolution Network for Monocular Depth Estimation

**DOI:** 10.3390/s26041199

**Published:** 2026-02-12

**Authors:** Wanting Jiang, Jun Li, Yaoqian Niu, Hao Chen, Shuang Peng

**Affiliations:** College of Electronic Science and Technology, National University of Defense Technology, Changsha 410073, China

**Keywords:** monocular depth estimation, lightweight network, structural reparameterization, CNNs

## Abstract

Monocular depth estimation (MDE) is a cornerstone task in 2D/3D scene reconstruction and recognition with widespread applications in autonomous driving, robotics, and augmented reality. However, existing state-of-the-art methods face a fundamental trade-off between computational efficiency and estimation accuracy, limiting their deployment in resource-constrained real-world scenarios. It is of high interest to design lightweight but effective models to enable potential deployment on resource-constrained mobile devices. To address this problem, we present RepACNet, a novel lightweight network that addresses this challenge through reparameterized asymmetric convolution designs and CNN-based architecture that integrates MLP-Mixer components. First, we propose Reparameterized Token Mixer with Asymmetric Convolution (RepTMAC), an efficient block that captures long-range dependencies while maintaining linear computational complexity. Unlike Transformer-based methods, our approach achieves global feature interaction with tiny overhead. Second, we introduce Squeeze-and-Excitation Consecutive Dilated Convolutions (SECDCs), which integrates adaptive channel attention with dilated convolutions to capture depth-specific features across multiple scales. We validate the effectiveness of our approach through extensive experiments on two widely recognized benchmarks, NYU Depth v2 and KITTI Eigen. The experimental results demonstrate that our model achieves competitive performance while maintaining significantly fewer parameters compared to state-of-the-art models.

## 1. Introduction

Monocular depth estimation (MDE)—the task of inferring dense depth maps from a single RGB image—is a fundamental problem in 2D/3D scene reconstruction and recognition with far-reaching applications in autonomous driving [1], robotics [2], augmented reality [3] and 3D reconstruction [4,5,6]. Unlike stereo vision [7] or LiDAR-based approaches [8] that exploit explicit geometric constraints, monocular depth estimation must infer 3D scene structure from inherently ambiguous 2D projections, relying on learned visual cues such as perspective, occlusion patterns, relative object scales, and texture gradients.

The field of monocular depth estimation has undergone significant evolution with advances in deep learning. Early CNN-based methods demonstrated that convolutional architectures could effectively extract semantic and geometric features [9,10,11,12] from images, establishing a strong foundation for dense prediction tasks. The robust feature extraction capability of CNNs substantially advanced monocular depth estimation [13,14]. However, traditional CNN architectures possess inherent limitations in capturing long-range dependencies due to their limited receptive fields [15]. This restriction constrains their ability to model global scene geometry—the spatial relationships and structures at image-level scales—which is crucial for accurate depth prediction in complex scenes with significant occlusions and large depth variations. To overcome this limitation, recent Transformer-based architectures [16,17,18] leveraged self-attention mechanisms to capture global context. These methods achieved notable accuracy improvements by enabling direct interaction between distant image regions without being constrained by convolutional receptive fields. Additionally, hybrid CNN-Transformer approaches [19,20] could balance receptive field and computational cost, achieving superior performance on standard benchmarks.

While Transformer-based and hybrid approaches have demonstrated impressive accuracy improvements, they incur substantial computational overhead and parameter counts [21]. The proliferation of mobile devices and edge computing applications has created an increasingly urgent demand for lightweight depth estimation systems that operate efficiently on resource-constrained hardware [22] while maintaining sufficient accuracy for safety-critical applications such as autonomous driving and robotic manipulation. This efficiency requirement has motivated the development of lightweight depth estimation methods [23,24].

Recognizing the efficiency challenge, several approaches have been proposed to develop lightweight depth estimation models [23,25,26,27,28,29]. Despite these diverse efforts, we found that there is still room for improvement in balancing accuracy and efficiency in these methods.

Recent architectural innovations in adjacent domains provide promising insights. First, MLP-Mixer architectures [30] have demonstrated that competitive global context modeling can be achieved through efficient spatial and channel mixing operations, avoiding the quadratic computational complexity of self-attention. Unlike Transformer-based approaches, MLP-Mixer achieves global feature interaction through linear complexity operations, making it fundamentally more scalable for resource-constrained scenarios. This suggests that global contexts do not require attention mechanisms. Second, structural reparameterization techniques [31] have shown that networks can effectively leverage multi-branch architectures during training—where diverse branch operations enhance feature learning—while consolidating these branches into a single efficient module during inference. This training-inference decoupling allows networks to maintain the representational capacity necessary for accurate predictions without sacrificing computational efficiency at deployment time. The results demonstrate that inference efficiency need not compromise training expressiveness. Together, these insights show a promising research direction: Designing a pure CNN-based architecture for monocular depth estimation that combines (1) efficient global context modeling inspired by MLP-Mixer and (2) training-inference architectural decoupling via structural reparameterization. Such an approach could potentially break the traditional efficiency-accuracy dilemma while maintaining explicit design principles tailored for monocular depth estimation.

To validate this hypothesis, we propose RepACNet, a pure CNN-based architecture specifically designed for efficient monocular depth estimation. Our approach introduces two complementary technical innovations. First, Reparameterized Token Mixer with Asymmetric Convolution (RepTMAC) captures long-range spatial dependencies through efficient spatial mixing operations inspired by MLP-Mixer [30], achieving global feature interaction with linear computational complexity rather than the quadratic overhead of self-attention. The design leverages structural reparameterization to maintain training-time expressiveness while ensuring inference efficiency. Second, Squeeze-and-Excitation Consecutive Dilated Convolutions (SECDCs) integrate adaptive channel attention with multi-scale dilated convolutions, enabling feature extraction that explicitly accounts for the multi-scale nature of depth variations across different scene regions. By strategically combining these innovations within a pure CNN backbone, RepACNet achieves competitive or superior performance compared to state-of-the-art methods while maintaining significantly fewer parameters and reduced computational demands, making it practical for deployment on mobile and edge devices. Our key contributions are as follows:

1. We propose RepACNet, a lightweight novel CNN-based network for monocular depth estimation, which can effectively address the current dilemma between accuracy and efficiency in the field of monocular depth estimation.
2. We design the RepTMAC module that efficiently captures long-range spatial dependencies through reparameterized asymmetric convolution operations, providing global context modeling without self-attention overhead. Additionally, the SECDC module, which integrates channel attention with dilated convolutions, is devised to extract depth-specific features across multiple receptive field sizes.
3. Comprehensive experiments on NYU Depth v2 [32] and KITTI Eigen [33] datasets demonstrate that RepACNet achieves competitive performance compared to state-of-the-art methods while maintaining significantly fewer parameters.

The remainder of this paper is organized as follows: Section 2 briefly reports the related works of MDE models, while Section 3 elaborates on the details of the proposed method. Experimental results are performed and discussed in Section 4. A discussion of this method’s limitations is provided in Section 5. Finally, the conclusion is given in Section 6.

## 2. Related Works

This section provides a comprehensive review of relevant literature, organized into three key areas that directly relate to our approach: lightweight monocular depth estimation, structural reparameterization techniques, and MLP-Mixer architectures in 2D/3D scene reconstruction and recognition.

### 2.1. Lightweight Monocular Depth Estimation

Traditional monocular depth estimation approaches [34,35] primarily depended on manually designed features and geometric cues, but these methods suffered from limited representational power and poor generalization capabilities. The advent of deep learning revolutionized this field, with CNN-based architectures delivering substantial performance improvements. Pioneering works by Eigen et al. [13,36], established the multi-scale prediction paradigm through coarse-to-fine refinement strategies, laying the groundwork for subsequent neural approaches. Laina et al. [14] advanced the field by introducing fully convolutional encoder–decoder architectures that better preserved spatial information during depth prediction.

The recent emergence of attention-based architectures [21,37,38] has opened new avenues for accuracy improvements in depth estimation. Nevertheless, the inherent quadratic scaling of attention computations presents severe deployment challenges for resource-constrained environments. Beyond achieving high accuracy, computational efficiency represents a critical design consideration for practical applications.

Motivated by lightweight design principles, researchers have developed specialized efficient architectures for depth estimation tasks. Early attempts like MiniNet [39] pursued aggressive parameter reduction strategies, achieving extremely compact models but often at the cost of significant accuracy degradation. More balanced approaches emerged with methods like LightDepthNet [28], which strategically applied depthwise separable convolutions [40,41] and channel reorganization techniques to achieve better efficiency-accuracy trade-offs. Multi-branch fusion strategies, as explored by Sui et al. [42], demonstrated how parallel lightweight feature extraction paths could be combined to improve both speed and estimation quality.

Contemporary lightweight depth estimation has witnessed increasingly sophisticated architectural innovations. Lite-Mono [26] represents a notable advancement in hybrid CNN-Transformer design, carefully balancing convolutional efficiency with selective transformer components to maintain competitive accuracy within constrained parameter budgets. Task-specific optimizations like those in ShuffleMono [27] have adapted channel shuffling strategies specifically for depth prediction requirements, showing how general lightweight principles can be customized for dense prediction tasks. Ultra-efficient approaches like Elite-Mono [43] have pushed parameter minimization to extreme levels for mobile deployment, while RepMono [29] has explored how structural reparameterization can decouple training complexity from inference efficiency.

Despite these significant advances, current lightweight depth estimation approaches continue to face fundamental limitations. Most existing methods achieve computational efficiency through architectural simplifications that inevitably constrain their feature learning capacity, resulting in suboptimal performance on geometrically complex scenes.

### 2.2. Structural Reparameterization Techniques

Structural reparameterization has emerged as a powerful technique for enhancing model performance during training while maintaining efficiency during inference. The concept was systematically introduced by Ding et al. [31] in RepVGG, which demonstrated how multi-branch training architectures could be algebraically collapsed into simpler inference-time structures. Their work showed that the representational benefits of complex training architectures could be preserved while eliminating runtime overhead through mathematical reparameterization.

Building on this foundation, numerous extensions have been proposed for different architectural components. RepVGG-style reparameterization has been adapted to various network components [44,45], including normalization layers, activation functions, and skip connections. Wang et al. [46] extended reparameterization to depthwise convolutions, showing how efficient mobile architectures could benefit from training time complexity without inference penalties. Similarly, Chen et al. [47] explored reparameterization in the context of neural architecture search, demonstrating how automated architecture design could leverage reparameterization principles.

The application of reparameterization to asymmetric convolutions has shown particular promise. Asymmetric convolutions, utilizing kernels of different shapes such as 1 × 3 and 3 × 1, have been shown to capture directional features effectively [48,49] while maintaining computational efficiency. Ding et al. [50] systematically studied how asymmetric convolution branches could be incorporated into reparameterizable frameworks, providing theoretical foundations for multi-branch asymmetric designs. Their analysis demonstrated that asymmetric kernels could capture complementary spatial patterns that improve overall feature learning quality.

Recent works have explored more sophisticated reparameterization strategies. Chandran et al. [51] investigated adaptive reparameterization where branch weights are learned during training, enabling more flexible feature combination strategies. Liu et al. [52] proposed hierarchical reparameterization that operates at multiple architectural levels, from individual layers to entire network blocks. Additionally, several works have studied the theoretical aspects of reparameterization, providing insights into when and why these techniques are effective.

However, existing reparameterization techniques still face several limitations. Most approaches focus on relatively simple architectural patterns and do not fully explore the potential of complex multi-branch designs for specific tasks like depth estimation. Furthermore, current reparameterization methods often do not consider the unique requirements of dense prediction tasks, where spatial feature patterns and multi-scale processing are particularly important.

### 2.3. MLP-Mixer Architectures

MLP-Mixer, introduced by Tolstikhin et al. [30], revolutionized the understanding of global feature mixing in 2D/3D scene reconstruction and recognition by demonstrating that simple multi-layer perceptrons could achieve competitive performance without attention mechanisms. The architecture’s core innovation lies in its dual-mixer design: token mixing for spatial feature interaction and channel mixing for feature transformation. This approach achieved remarkable results on image classification tasks while maintaining linear computational complexity, challenging the dominance of attention-based methods.

Several works have explored improvements to the original MLP-Mixer design. FNet [53] replaced attention mechanisms in transformers with Fourier transforms, showing that global mixing could be achieved through various mathematical operations. gMLP [54] introduced gating mechanisms to enhance the selectivity of token mixing operations, improving performance on challenging visual recognition tasks. S^2^-MLP [55] proposed spatial-shift operations as an alternative to linear token mixing, demonstrating that simple geometric transformations could provide effective spatial feature interaction.

Recent research has investigated convolutional adaptations of MLP-Mixer principles. ConvMixer [56] replaced linear layers with depthwise convolutions, maintaining the dual-mixer paradigm while leveraging spatial inductive biases inherent in convolution operations. Their approach showed that convolutional token mixing could provide better performance for vision tasks compared to purely linear alternatives. WaveMix [57] explored wavelet-based token mixing, demonstrating how frequency domain operations could enhance spatial feature interaction in mixer architectures.

The adaptation of MLP-Mixer to dense prediction tasks has also been explored. CycleMLP [58] introduced cycle-consistent token mixing for improved spatial feature propagation in dense prediction scenarios. SegMixer [59] specifically adapted mixer architectures for semantic segmentation, showing how token mixing could be optimized for pixel-level classification tasks. However, these adaptations typically focused on high-level semantic tasks rather than geometric understanding tasks like depth estimation.

Despite the promising developments in MLP-Mixer architectures, several limitations persist in their application to depth estimation tasks. Most existing adaptations retain the original linear token mixing approach, which may not optimally leverage the spatial structure and geometric relationships crucial for depth perception. The integration of mixer principles with convolutional architectures for dense prediction tasks remains underexplored, particularly for applications requiring fine-grained spatial understanding. Furthermore, current mixer architectures often lack effective multi-scale processing capabilities, limiting their ability to capture the diverse spatial patterns essential for robust depth estimation across different object scales and distances.

## 3. Method

### 3.1. Design Motivation and Choice

**Figure 1:**
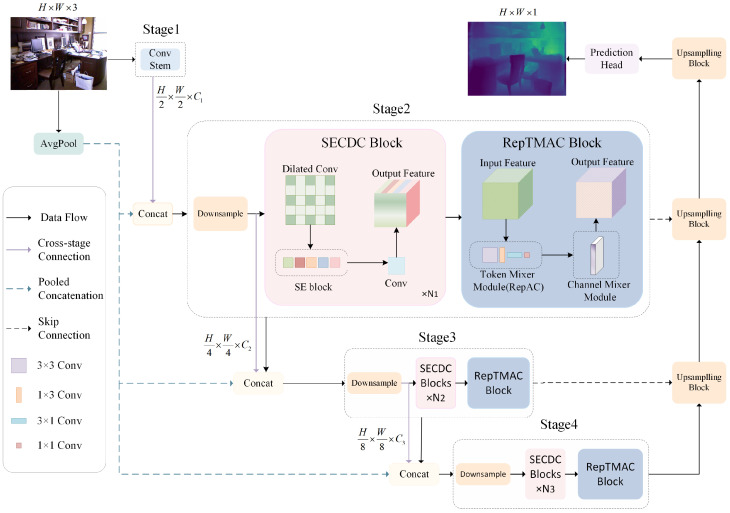
Overview of the proposed RepACNet. The encoder consists of four stages, and it uses Squeeze-and-Excitation Consecutive Dilated Convolution (SECDC) modules and Reparameterized Token Mixer with Asymmetric Convolution (RepTMAC) modules to extract rich hierarchical features. The black solid lines represent data flow, the purple solid lines indicate cross-stage connections, the blue dashed lines represent pooled concatenation, and the black dashed lines denote skip connections. The details of these modules are shown in Figure 2 and Figure 3.

Monocular depth estimation has witnessed significant advancement with the development of CNN-based encoder–decoder architectures. However, current approaches face a fundamental trade-off between model accuracy and computational efficiency. While deeper networks with larger receptive fields can capture more contextual information for accurate depth prediction, they inevitably introduce substantial computational overhead that limits their deployment in resource-constrained environments. This motivates our design of RepACNet, as shown in Figure 1, a lightweight yet effective architecture specifically tailored for monocular depth estimation.

**Enhanced Local Feature Extraction.** Traditional CNN architectures suffer from limited receptive fields, particularly in shallow networks designed for efficiency. To address this limitation, we propose the SECDC module. Unlike conventional dilated convolutions, SECDC integrates Squeeze-and-Excitation attention mechanisms directly after the depth-wise dilated convolution [26] and before channel transformation. This design allows the network to selectively emphasize important spatial features while expanding the receptive field through Consecutive Dilated Convolutions with varying dilation rates. The SE attention mechanism provides channel-wise recalibration, enabling the model to focus on the most informative features for depth estimation without introducing significant computational cost. Table 1 shows more details.

**Figure 4:**
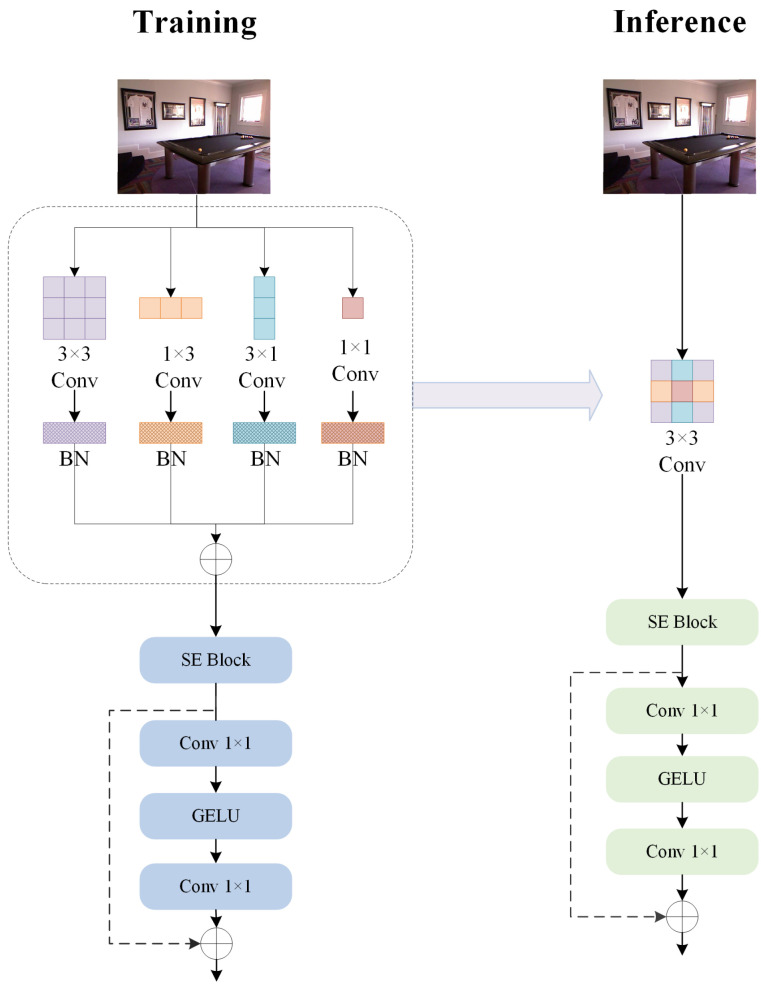
Equivalent diagram of RepTMAC module structure. Where the multi-branch structure (3 × 3, 1 × 1, 1 × 3, and 3 × 1) during training is converted to a single-branch (3 × 3) structure during inference.

**Efficient Global Context Modeling.** While SECDC effectively captures local features with expanded receptive fields, global context modeling remains crucial for accurate depth estimation. However, traditional self-attention mechanisms incur quadratic computational complexity with respect to spatial resolution, making them unsuitable for lightweight architectures. To overcome this challenge, we introduce the RepTMAC. RepTMAC consists of two key components: a RepAC module for spatial token mixing, and efficient channel mixing through 1 × 1 convolutions. The RepAC module leverages four parallel convolutional branches (3 × 3, 1 × 1, 1 × 3, and 3 × 1) during training to capture multi-scale spatial patterns, which are then reparameterized into a single 3 × 3 convolution during inference for computational efficiency. It is shown in Figure 4. This design enables the model to capture both local and global features effectively while maintaining linear computational complexity. The Channel Mixer employs inverted bottleneck residuals with GELU activation to enhance inter-channel information exchange.

### 3.2. Network Architecture

Our RepACNet follows an encoder–decoder architecture where the encoder employs a three-stage design with progressively increasing feature dimensions. Each stage combines multiple SECDC modules for local feature extraction followed by a single RepTMAC module for global context aggregation. This hierarchical design ensures that both fine-grained local details and coarse-grained global context are effectively captured at multiple scales. The decoder utilizes skip connections with channel attention mechanisms to preserve high-frequency details during upsampling, enabling accurate depth boundary prediction.

The proposed architecture achieves an effective balance between computational efficiency and depth estimation accuracy by our lightweight yet effective SECDC and RepTMAC modules, while maintaining the representational power necessary for high-quality monocular depth estimation. Figure 1 shows the proposed architecture. Conv Stem consists of three convolutions: first, it goes through a 3 × 3 downsampling layer, and then through two additional 3 × 3 convolutions with stride=1 for local feature extraction.

**Depth encoder.** Our encoder follows a multi-stage design that effectively balances feature extraction capability with computational efficiency. The input RGB image with dimensions H×W×3 is first processed by a convolution stem consisting of three consecutive 3 × 3 convolutions. The first convolution applies stride = 2 to downsample the input, generating initial feature maps of size $\frac{H}{2}\times \frac{W}{2}\times C_{1}$. The subsequent two convolutions with stride = 1 perform local feature refinement while maintaining spatial resolution. Unlike conventional approaches that only use features from the previous stage, our architecture concatenates features from multiple sources: the output of the previous stage after downsampling, the previous stage’s final output, and average-pooled versions of the original input image at different scales. This concatenation strategy, formulated as

(1) $$X^{\left(i\right)}=Concat\left(F^{\left(i-1\right)},{Pool}^{\left(i-1\right)}\left(I\right),F_{prev}^{\left(i-1\right)}\right)$$

where $F^{\left(i-1\right)}$ represents features from the previous stage, $Pool^{\left(i-1\right)}\left(I\right)$ denotes the average-pooled input image, and $F_{prev}^{\left(i-1\right)}$ is the accumulated feature from earlier stages. This design compensates for spatial information loss during downsampling and provides rich multi-scale context for subsequent processing. Each stage employs a carefully designed sequence of our proposed modules. The majority of each stage consists of SECDC modules, followed by a single RepTMAC module for global context aggregation. The specific configuration follows the pattern [(depth[i]−1)×SECDC,1×RepTMAC] for each stage *i*, where depth = [4, 4, 10].

**SE-enhanced Consecutive Dilated Convolutions.** The SECDC module extends traditional dilated convolutions by integrating channel attention for enhanced feature selection. Given an input feature *X* with dimension H×W×C, the SECDC module processes features as follows:

(2) $$X_{dilated}=DDWConv_{i}\left(X\right)$$

(3) $$X_{bn}=BN\left(X_{dilated}\right)$$

(4) $$X_{attended}=X_{bn}⊙SE\left(X_{bn}\right)$$

(5) $$X_{out}=X+DropPath\left(Linear_{G}\left(Linear\left(LN\left(X_{attended}\right)\right)\right)\right)$$

where $DDWConv_{r}$ denotes depth-wise dilated convolution with dilation rate r, BN is a batch normalization layer, SE represents the Squeeze-and-Excitation attention mechanism, and ⊙ indicates element-wise multiplication.

**Reparameterized Token Mixer with Asymmetric Convolutions (RepTMAC).** Although RepTMAC is purely convolutional, it achieves global-like context modeling inspired by MLP-Mixer [30]. By separating spatial mixing (via RepAC) and channel mixing, and stacking multiple blocks, RepTMAC enables information to propagate across the entire spatial domain. The asymmetric kernels (1×3,3×1) further enhance the capture of anisotropic long-range dependencies (e.g., walls, roads), effectively simulating the global attention capability of Transformers with linear computational complexity. RepTMAC serves as the global context aggregation module, replacing computationally expensive self-attention mechanisms with efficient convolutional operations. The module consists of two main components: the RepAC Token Mixer and the Channel Mixer. The RepAC Token Mixer employs four parallel convolutional branches during training:

(6) $$X_{3\times 3}=Conv_{3\times 3}^{dw}\left(X;W_{3\times 3}\right)$$

(7) $$X_{1\times 1}=Conv_{1\times 1}^{dw}\left(X;W_{1\times 1}\right)$$

(8) $$X_{1\times 3}=Conv_{1\times 3}^{dw}\left(X;W_{1\times 3}\right)$$

(9) $$X_{3\times 1}={Conv}_{3\times 1}^{dw}\left(X;W_{3\times 1}\right)$$

(10) $$X_{token}=BN\left(X_{3\times 3}\right)+BN\left(X_{1\times 1}\right)+BN\left(X_{1\times 3}\right)+BN\left(X_{3\times 1}\right)$$

where $W_{3\times 3}$, $W_{1\times 1}$, $W_{1\times 3}$ and $W_{3\times 1}$ are the learnable weight parameters of the 3 × 3, 1 × 1, 1 × 3, and 3 × 1 depth-wise convolutional kernels, respectively. These parameters are learned through standard backpropagation during training, allowing each branch to specialize in capturing different global and local features. The structural reparameterization is mathematically viable due to the linearity of convolution. During inference, utilizing the additivity of convolution, the trained weights from all four branches are merged into a single 3×3 kernel $W_{fused}$ to reduce computational cost without losing representational capacity [31]. The derivation is formulated as

(11) $$W_{fused}=W_{3\times 3}+{Pad}_{3\times 3}\left(W_{1\times 1}\right)+{Pad}_{3\times 3}\left(W_{1\times 3}\right)+{Pad}_{3\times 3}\left(W_{3\times 1}\right)$$

(12) $$b_{fused}=b_{3\times 3}+b_{1\times 1}+b_{1\times 3}+b_{3\times 1}$$

where $Pad_{3\times 3}\left(\cdot \right)$ denotes zero-padding operations to align all kernels to 3 × 3 dimensions, and b represents the bias terms. This reparameterization preserves the representational capacity learned during training while eliminating the computational overhead of parallel branches during inference. The Channel Mixer follows an inverted bottleneck design:

(13) $$X_{channel}=X_{token}+DropPath\left(Conv_{1\times 1}\left(GELU\left(Conv_{1\times 1}\left(X_{token}\right)\right)\right)\right)$$

where the hidden dimension expansion ratio is set to 2 (i.e., the intermediate layer has 2× the input channels) for balanced performance and efficiency.

**depth Decoder.** The decoder employs a hierarchical upsampling strategy to reconstruct depth maps from the encoded features. Following the design principles of efficient depth estimation networks, we use bilinear upsampling for spatial dimension recovery combined with skip connections to preserve fine-grained details. The decoder processes features from three encoder stages (scales [0, 1, 2]) through consecutive upsampling blocks. Each upsampling block consists of the following:

- Initial feature processing:
  (14) $$X=Conv_{3\times 3}\left(F_{enc}^{\left(i\right)}\right)$$
  (where $F_{enc}^{\left(i\right)}$ represents features from the encode stage);
- Bilinear upsampling:
  (15) $$X_{up}=Upsample\left(X\right);$$
- Skip connection fusion:
  (16) $$Concat(X_{up},F_{skip}^{\left(i-1\right)})$$
  (where $F_{skip}^{\left(i-1\right)}$ represents features from the previous encode stage, only the first and second stage have skip connection fusion);
- Feature refinement:
  (17) $$Conv_{3\times 3}\left(X_{concat}\right).$$

The final depth prediction is generated through a 1 × 1 convolution followed by sigmoid activation and scaling:

(18) $$D=\sigma \left(Conv_{3\times 3}\left(X_{final}\right)\right)\times D_{max}$$

where σ denotes the sigmoid function and $D_{max}$ represents the maximum depth value for the specific dataset.

### 3.3. Training Loss

Following established practices in monocular depth estimation [13,21,60,61], we employ the Scale-Invariant Logarithmic (SILog) loss to supervise our RepACNet training. The SILog loss is particularly suitable for depth estimation as it focuses on relative depth relationships rather than absolute values, making the model robust to global scale variations that are inherent in monocular depth prediction. Given ground-truth depth $d_{i}^{*}$ and predicted depth ${\hat{d}}_{i}$ at pixel *i*, we first compute the logarithmic residual:

(19) $$\Delta d_{i}=\log{\hat{d}}_{i}-\log d_{i}^{*}$$

For *K* pixels with valid depth values (determined by the mask), the scale-invariant loss is formulated as

(20) $$L_{SILog}=\alpha \sqrt{\left(1/K\right)\sum_{i}\Delta d_{i}^{2}-\lambda \left(1/K^{2}\right){\left(\sum_{i}\Delta d_{i}\right)}^{2}}$$

where λ is the variance focus parameter that controls the emphasis on variance minimization, and α is a scaling constant. The first term captures the mean squared logarithmic error, while the second term reduces the impact of systematic scale bias by penalizing the variance of residuals. In our implementation, we set λ = 0.85 following previous lightweight depth estimation works [21,62], which provides an effective balance between accuracy and scale invariance. The scaling factor α = 10.0 is applied to bring the loss magnitude to a suitable range for optimization. Only pixels with valid depth measurements (mask = True) contribute to the loss computation, ensuring that the model is not penalized for predictions at invalid depth locations.

The SILog loss design aligns well with our lightweight architecture philosophy, as it provides effective supervision without requiring complex multi-scale loss computations or additional regularization terms, maintaining both training efficiency and depth estimation quality.

## 4. Experiments

This section evaluates the proposed framework and demonstrates the superiority of RepACNet. In this section, we compare our method with several baselines. Results are cited from original papers, except for methods with unavailable source code or differing resolutions, which were re-implemented and retrained under the same setting for fair comparison.

### 4.1. Datasets

**NYU Depth V2 dataset.** NYU Depth v2 is a dataset that provides images and depth maps for different indoor scenes captured at a pixel resolution of 640 × 480 [32]. It is an indoor datasets with 120K RGB-D videos captured from 464 indoor scenes. The depth maps have an upper bound of 10 m. Our network outputs depth prediction having a resolution the same as the dataset. Following previous work [62], we use 24,231 images for training and 654 images for testing.

**KITTI dataset.** The KITTI dataset [33] serves as a widely adopted benchmark for monocular depth estimation in autonomous driving scenarios, containing outdoor driving sequences captured from a moving vehicle equipped with stereo cameras, 3D LiDAR, and GPS/IMU sensors. For monocular depth estimation evaluation, we follow the standard Eigen split [13] protocol, which partitions the dataset into 23,488 training image pairs and 697 testing images with corresponding LiDAR-derived ground-truth depth maps. The dataset captures diverse driving conditions, including urban streets, highways, and residential areas, providing realistic scenarios for evaluating lightweight depth estimation models intended for autonomous vehicle deployment. We restrict the predicted depth range to [0, 80] m in accordance with the standard KITTI depth estimation protocol, which aligns with the practical sensing range requirements for autonomous driving applications.

### 4.2. Implementation Details

RepACNet employs a two-stage training strategy consisting of ImageNet pretraining followed by depth estimation fine-tuning.

**Pretraining Stage:** The backbone encoder is first pre-trained on the ImageNet-1K classification task for 300 epochs using the AdamW optimizer with a learning rate of $1\times 10^{-3}$, a weight decay of 0.05, and cosine learning rate scheduling with a 5-epoch warmup. The input resolution is set to 256 × 256 with standard ImageNet augmentations, including AutoAugment, color jittering, and random erasing. This pretraining phase enables the RepACNet to learn robust multi-scale feature representations on large-scale natural images before specializing for depth estimation.

**Figure 5:**
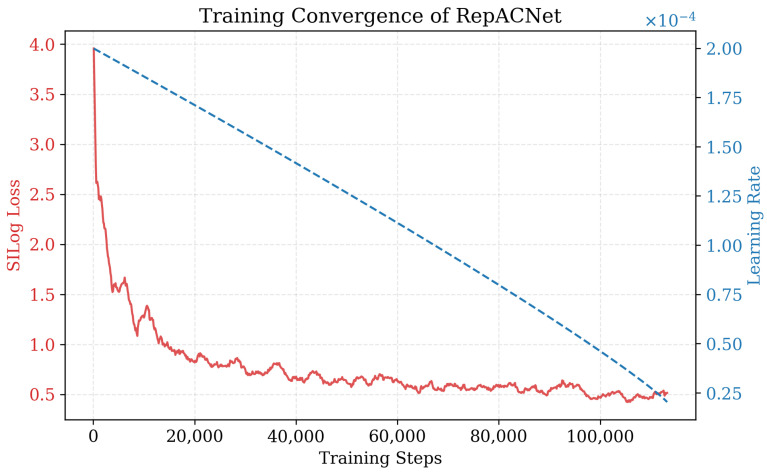
Training convergence analysis of RepACNet. The red solid line indicates the SILog loss, which decreases consistently and stabilizes, demonstrating the effective convergence of our model. The blue dashed line represents the learning rate schedule.

**Fine-tuning Stage:** The pretrained encoder is then integrated into RepACNet and fine-tuned for monocular depth estimation. RepACNet is implemented in PyTorch (version 1.10.0) and trained on NVIDIA RTX 4090 GPUs. We use the Adam optimizer with an initial learning rate of $2\times 10^{-4}$, a weight decay of $1\times 10^{-2}$, and a batch size of 16. The model is trained for 50 epochs with polynomial learning rate decay (power = 0.9). During training, we employ standard data augmentation strategies to prevent overfitting. Specifically, random horizontal flipping is applied synchronously to both the input RGB image and the ground truth depth map with a probability of 0.5. This ensures geometric consistency is maintained. Additionally, we apply random color jittering (gamma, brightness, and color shifts) to the RGB images, following the protocol in [36]. The training follows the standard protocols for each dataset, with depth range restrictions of [0, 80] m for KITTI and [0, 10] m for NYU v2 during evaluation. The training curve of RepACNet is as in Figure 5.

The ImageNet pretraining provides crucial initialization for RepACNet, enabling them to capture diverse directional and scale-specific patterns that transfer effectively to depth estimation tasks while maintaining computational efficiency during inference.

### 4.3. Evaluation Metrics

We follow the standard evaluation protocols established for monocular depth estimation to ensure a fair comparison with existing methods. Our evaluation employs a comprehensive set of metrics that capture different aspects of depth prediction quality, including both accuracy and error measurements. Our evaluation employs dataset-specific metric combinations that best reflect the characteristics and requirements of indoor versus outdoor depth estimation scenarios.

**Accuracy Metrics:** We adopt the widely-used threshold metrics [36] to measure the percentage of pixels with acceptable relative error:

(21) $$\mathrm{Accuracy}=\frac{1}{N}\sum_{i=1}^{N}I\left(\max\left(\frac{d_{i}}{d_{i}^{*}},\frac{d_{i}^{*}}{d_{i}}\right)<thr\right)$$

where I(·) is the indicator function, $d_{i}$ is the predicted depth, $d_{i}^{*}$ is the ground truth, *N* is the total number of valid pixels, and thr denotes the threshold. We denote this metric as δ. Specifically: $\delta_{1}$ (threshold = 1.25) indicates the percentage of pixels with prediction ratio within [0.8, 1.25]; $\delta_{2}$ (threshold = $1.25^{2}$ = 1.56) indicates the percentage within [0.64, 1.56] and $\delta_{3}$ (threshold = $1.25^{3}$ = 1.95) indicates the percentage within [0.51, 1.95].

**Dataset-Specific Error Metrics:**

(1) NYU Depth v2: Following the standard indoor evaluation protocol, we report AbsRel (Absolute Relative Error); mean relative error $\frac{\left|d_{gt}-d_{pred}\right|}{d_{gt}}$, *log*_10_; mean absolute error in the $log_{10}$ space, $mean(\left|\log_{10}\left(d_{gt}\right)-\log_{10}\left(d_{pred}\right)\right|)$, emphasizing multiplicative rather than additive errors; RMSE (Root Mean Square Error), $\sqrt{mean({\left(d_{gt}-d_{pred}\right)}^{2}})$ in meters; and Params, model parameter count in millions, crucial for assessing deployment feasibility.
(2) KITTI Eigen Split: Following the standard outdoor driving evaluation protocol, we report AbsRel and SqRel (Squared Relative Error) and mean squared relative error $\frac{{\left(d_{gt}-d_{pred}\right)}^{2}}{d_{gt}}$, providing a heavier penalty for large errors critical in driving scenarios, RMSE and Params.

**Parameter Efficiency:** We consistently report Params across both datasets to evaluate the efficiency-accuracy trade-off, which is central to our lightweight architecture design. This metric directly reflects deployment feasibility on resource-constrained devices, making it essential for fair comparison among lightweight depth estimation methods.

### 4.4. Comparison to the State-of-the-Art

**Figure 6:**
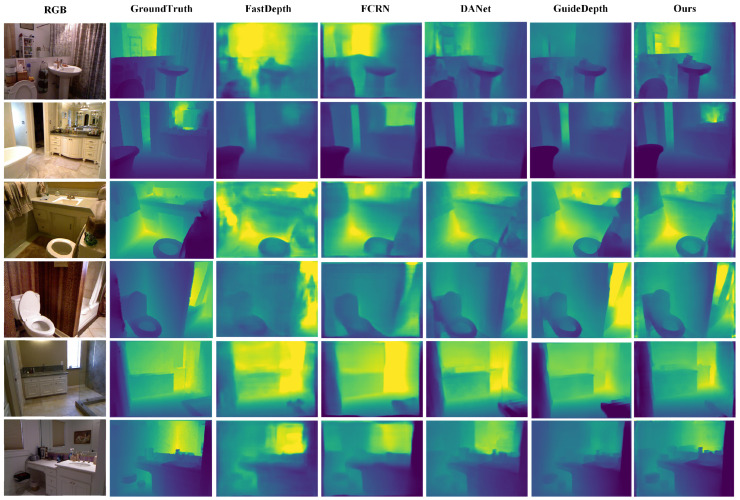
Qualitative results on the NYU Depth v2 dataset.

**Figure 7:**
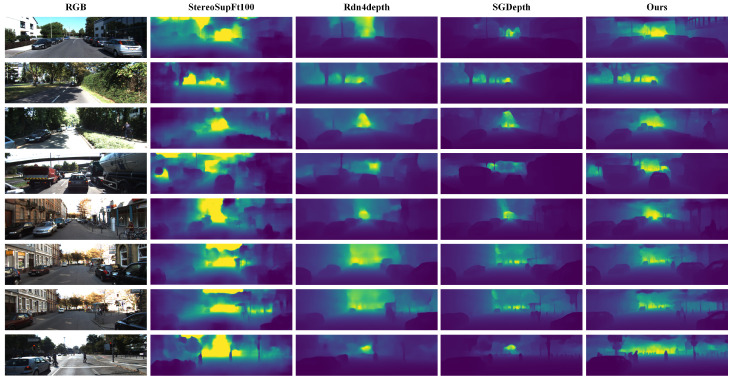
Qualitative results on the Eigen split of KITTI dataset.

Our proposed RepACNet achieves competitive results that demonstrate both superior accuracy and notable parameter efficiency compared to state-of-the-art methods. We evaluate RepACNet comprehensively on both the NYU Depth v2 and KITTI datasets, providing quantitative comparisons in Table 2 and Table 3, along with qualitative analyses in Figure 6 and Figure 7. In the depth maps, colors represent depth values, with blue indicating closer objects and red representing more distant ones.

**NYU Depth v2 Quantitative Results:** As shown in Table 2, RepACNet achieves highly competitive performance on the indoor dataset. Our method attains $\delta_{1}$ = 0.852, $\delta_{2}$ = 0.974, and $\delta_{3}$ = 0.995, demonstrating meaningful improvements in accuracy across all precision thresholds. Notably, while CaBins [63] achieves slightly higher $\delta_{1}$ accuracy (0.860), it requires 181.3 M parameters—over 64× more than our 2.8 M parameter model. Compared to other lightweight methods, RepACNet shows substantial improvements over existing efficient methods. Compared to FastDepth [64], our method achieves a $\delta_{1}$ improvement of 0.050 (0.852 vs. 0.802) while maintaining comparable parameter efficiency (2.8 M vs. 3.9 M). Additionally, RepACNet surpasses GuideDepth [25] by 0.029 in $\delta_{1}$ accuracy (0.852 vs. 0.823) with fewer parameters (2.8 M vs. 5.8 M). Notably, our model outperforms LightNet [23] with significant improvements of 0.070 in $\delta_{1}$ (0.852 vs. 0.782) while using substantially fewer parameters (2.8 M vs. 16 M). In terms of error metrics, RepACNet achieves competitive Log10 (0.053) and RMSE (0.423) values, trailing only DORN [65] in Log10 performance but maintaining superior parameter efficiency.

**Table 2:** Quantitative comparison results of RepACNet with some recent representative methods on the NYU Depth v2 benchmark. The symbols “↑/↓” mean that a higher/lower value is better. ∗ indicates the use of ResNet.

Method	$\delta_{1}↑$	$\delta_{2}$ ↑	$\delta_{3}$ ↑	*log*_10_ ↓	RMSE ↓	Abs Rel ↓	Params (M) ↓
Eigen et al. [36]	0.769	0.950	0.988	—	0.641	0.158	141
FCRN [14]	0.811	0.953	0.988	0.055	0.573	0.127	64
DCNF [66]	0.702	0.921	0.979	0.074	0.722	0.196	40
Xu et al. [67]	0.806	0.952	0.986	0.057	0.593	0.125	27
DORN [65] ∗	0.832	0.971	0.992	**0.051**	0.501	**0.115**	381
FastDepth [64]	0.802	0.950	0.982	0.061	0.580	0.157	3.9
Wu et al. [68]	0.805	0.966	0.993	—	0.531	0.132	112
DANet [69]	0.839	0.969	0.993	0.055	0.482	0.130	8.2
GuideDepth [25]	0.823	0.961	0.990	0.058	0.501	0.138	5.8
Lite-mono [26]	0.762	0.939	0.986	0.069	0.539	0.167	3.1
CaBins [63]	**0.860**	**0.974**	0.994	0.054	**0.419**	0.120	181.3
LightDepthNet [28]	0.838	—	—	—	0.457	—	**2.6**
LightNet [23]	0.782	0.956	0.991	0.062	0.510	0.151	16
**Ours**	0.852	**0.974**	**0.995**	0.053	0.423	0.123	2.8

The best and the second best results are highlighted in bold and underlined, respectively.

**KITTI Quantitative Results:** Table 3 presents the quantitative comparison results on the KITTI Eigen split. RepACNet achieves state-of-the-art performance across all reported metrics, securing the best results with $\delta_{1}=0.961$, $\delta_{2}=0.995$, and $\delta_{3}=0.999$. Remarkably, our method outperforms the recent heavy-weight model CaBins [63] in terms of both accuracy ($\delta_{1}$: 0.961 vs. 0.959) and error rates (RMSE: 2.324 vs. 2.332), while utilizing approximately 65× fewer parameters (2.8 M vs. 181.3 M). When compared to representative lightweight approaches, RepACNet demonstrates a significant performance advantage. Specifically, it surpasses Lite-Mono [26] with a $\delta_{1}$ improvement of 0.033 (0.961 vs. 0.928) and achieves considerable reductions in error metrics (Abs Rel: 0.060 vs. 0.076). These results indicate that RepACNet attains a superior trade-off between depth estimation accuracy and parameter efficiency.

**Table 3:** Quantitative comparison results of RepACNet with some recent representative methods on the Eigen split of KITTI dataset. ∗ indicates the use of ResNet, ⋆ indicates self-supervised methods, ⋇ indicates unsupervised learning. The symbols “↑/↓” mean that a higher/lower value is better.

Method	$\delta_{1}$ ↑	$\delta_{2}$ ↑	$\delta_{3}$ ↑	Sq Rel ↓	RMSE ↓	Abs Rel ↓	Params (M) ↓
DCNF [66]	0.656	0.881	0.958	—	7.046	0.217	40
DORN [65] ∗	0.940	0.989	0.994	0.301	2.719	0.066	381
StereoSupFt100 [70]	0.892	0.969	0.987	0.652	4.156	0.095	159
rdn4depth [71] ⋇	0.844	0.953	0.978	0.899	4.352	0.134	66.9
SGDepth [72] ⋆	0.881	0.959	0.981	0.885	4.698	0.117	15
Wu et al. [68]	0.901	0.977	0.992	—	3.248	0.092	134
GuideDepth [25]	0.813	0.950	0.986	—	4.942	0.135	5.8
P3Depth [73]	0.953	0.993	0.998	0.270	2.842	0.071	48
Lite-Mono [26] ⋆	0.928	0.986	0.997	0.330	2.960	0.076	3.1
Sui et al. [42] ⋆	0.905	0.969	0.988	0.498	3.407	0.104	3
CaBins [63]	0.959	0.991	0.997	0.203	2.332	0.063	181.3
Repmono [29] ⋆	0.897	0.985	0.983	0.729	4.445	0.101	**2.31**
LightNet [23]	0.940	0.991	0.998	0.283	2.813	0.071	16
**Ours**	**0.961**	**0.995**	**0.999**	**0.194**	**2.324**	**0.060**	2.8

The best and the second best results are highlighted in bold and underlined, respectively.

**Qualitative Analysis:** Figure 6 illustrates RepACNet’s depth estimation quality on indoor scenes. Compared to FastDepth [64], FCRN [14], and GuideDepth [25], our method produces more accurate depth boundaries around furniture, clearer spatial relationships between objects, and better preservation of fine details in complex indoor environments (NYU Depth v2). The depth maps show well-defined contours of tables, chairs, and room structures with smooth depth transitions.

Similarly, Figure 7 illustrates RepACNet’s depth estimation performance on outdoor driving scenarios (KITTI), where our method generates depth maps with clear hierarchical representations of vehicles, pedestrians, road infrastructure, and background elements. The depth estimation maintains high fidelity across varying lighting conditions and scene complexities, crucial for autonomous driving applications.

**Figure 8:**
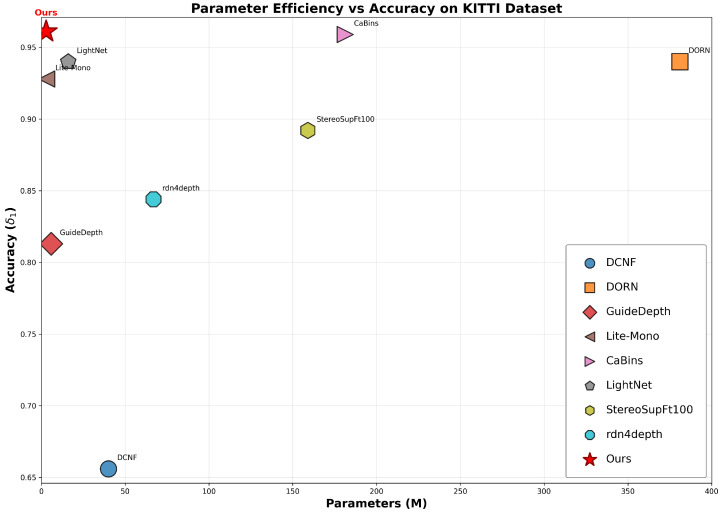
Complextity and performance comparisons of representative lightweight depth estimation methods.

**Parameter Efficiency Achievement:** Most importantly, RepACNet achieves this superior performance while requiring only 2.8 M parameters, making it one of the most parameter-efficient depth estimation networks. This represents a significant advance in deployment feasibility for resource-constrained environments, achieving accuracy levels comparable to methods requiring 10–65× more parameters, which is shown in Figure 8.

### 4.5. Ablation Study

To validate the effectiveness of each component in RepACNet, we conduct comprehensive ablation experiments on the NYU Depth v2 dataset. As shown in Table 4, we systematically evaluate the impact of different configurations in overall architecture design on model performance. The proposed RepACNet achieves a superior trade-off with only 5.6 GFLOPs and 2.8 M parameters. Considering that modern mobile processors (e.g., Apple A-series or Qualcomm Snapdragon) offer computational capacities in the order of TOPS (Tera Operations Per Second), our model’s low complexity theoretically ensures real-time inference capability on edge devices. We do not report on-device latency measurements; this statement is based on the complexity analysis.

**SECDC Module Analysis:** Removing the SE attention mechanism from the Consecutive Dilated Convolution leads to noticeable performance degradation, with $\delta_{1}$ from 0.852 dropping to 0.826 and AbsRel increasing to 0.131. This indicates that the Squeeze-and-Excitation mechanism plays a crucial role in channel-wise feature recalibration, particularly important for capturing fine-grained depth information.

**RepAC Effectiveness:** To test the effectiveness of RepAC, we replaced the four-branch structure (3 × 3, 1 × 1, 1 × 3, and 3 × 1 depth-wise convolutional) in RepTMAC with a residual branch, a 1 × 1 branch, and a 3 × 3 branch. Replacing branches results in substantial performance drops: $\delta_{1}$ decreases from 0.837 to 0.778, AbsRel increases from 0.127 to 0.148, and RMSE rises from 0.444 to 0.519. This demonstrates the critical importance of incorporating asymmetric convolutions in the Token Mixer for effective feature representation.

**Skip Connections Analysis:** Removing skip connections from the decoder reduces the parameter count to 2.7 M while maintaining stable performance ($\delta_{1}$ = 0.841, AbsRel = 0.126). The results show that skip connections have minimal impact on performance in our lightweight architecture, suggesting that the encoder features contain sufficient information for accurate depth prediction.

**RepTMAC Analysis:** To demonstrate the importance of the RepTMAC component, we replaced RepTMAC with a standard 3 × 3 dilated convolution. The architecture (without RepTMAC components) increases the parameter count to 3.0 M but shows substantial performance degradation, with $\delta_{1}$ dropping from 0.837 to 0.791 and AbsRel increasing from 0.127 to 0.148. This comparison clearly demonstrates the advantage of RepACNet’s MLP-Mixer design, achieving superior performance with fewer parameters (2.8 M vs. 3.0 M).

These comprehensive ablation studies validate the effectiveness of each proposed component and demonstrate that RepACNet achieves an optimal balance between accuracy and computational efficiency for lightweight monocular depth estimation.

## 5. Discussion

**Figure 9:**
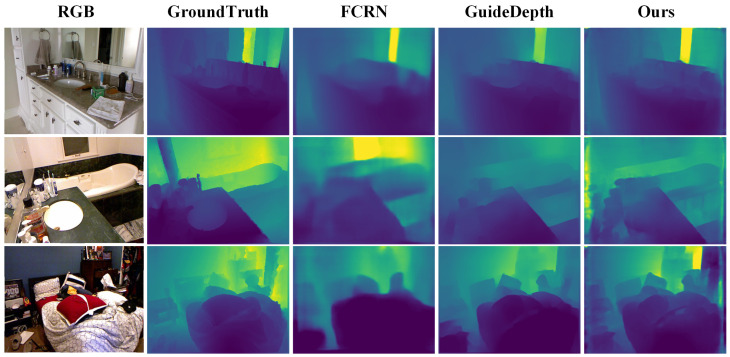
Failure cases of the proposed RepACNet method and other advanced methods.

Although RepACNet achieves competitive performance on standard benchmarks, our method exhibits notable limitations when handling complex real-world scenarios. As illustrated in Figure 9, the method particularly struggles in cluttered indoor scenes with multiple small objects at similar depths. However, we have noticed that this is also challenging for other advanced methods.

The primary limitations stem from two intrinsic characteristics of our lightweight design:

(1) **CNN Smoothing Bias:** Pure convolutional architectures naturally act as low-pass filters, tending to smooth out high-frequency signals. This results in blurred depth predictions at sharp edges (e.g., table legs or chair backs). Similar challenges in detecting structural discontinuities are also observed in other domains, such as network vector autoregression models [74], highlighting the universal difficulty of boundary detection without explicit edge modeling.
(2) **Lack of Explicit Semantic Guidance:** Unlike methods integrating semantic segmentation heads or Transformer-based global attention, RepACNet relies primarily on local texture and structural cues. In cluttered scenes with similar textures at varying depths (as shown in Figure 9), the model struggles to infer correct spatial relations without high-level semantic context to distinguish between objects.

To address these limitations, we identify several promising research directions:

(1) Spatial Attention Integration: Extending SECDC with spatial attention mechanisms would enable the network to learn explicit object boundaries. A CBAM-style extension combining channel and spatial attention could suppress smooth regions while preserving depth discontinuities at object edges, with minimal computational overhead.
(2) Boundary-Aware Loss Functions: Augmenting the training objective with edge-preserving terms would directly encourage sharp depth predictions at object boundaries. For instance, adding a gradient-matching loss that penalizes discrepancies in depth gradients between predictions and ground truth could enhance boundary sharpness RMSE without architectural modifications.
(3) Multi-Task Learning with Semantic Guidance: Incorporating semantic segmentation as an auxiliary task would provide scene understanding that naturally guides depth prediction. Different semantic classes inherently require distinct depth values, which would force the network to learn sharper boundaries. This approach, inspired by existing semantic-guided methods, could yield RMSE improvements in cluttered scenes.
(4) Adaptive Receptive Fields: Employing larger dilation rates or deformable convolutions would enhance the model’s ability to capture complete object structures rather than local spatial patterns. This would improve multi-object reasoning and scene understanding at the object scale.
(5) Instance-Level Depth Reasoning: A more fundamental approach would extend the architecture to perform instance-aware depth prediction, where the network reasons about individual object instances rather than purely pixel-wise depth. This could involve lightweight object detection alongside depth estimation, allowing the model to assign depth values with explicit object-instance awareness.

Among these directions, we recommend prioritizing spatial attention integration and boundary-aware losses, as they offer substantial improvements with minimal architectural changes. Multi-task learning with semantic guidance represents a medium-term direction with significant potential. More sophisticated approaches like instance-level reasoning would constitute longer-term research opportunities.

## 6. Conclusions

This paper presents RepACNet, a novel lightweight pure CNN architecture for monocular depth estimation that achieves superior parameter efficiency while maintaining high accuracy. The proposed design incorporates the innovative RepTMAC module, which effectively captures multi-scale spatial features through its multi-branch asymmetric convolution structure without relying on attention mechanisms. Experimental results on the KITTI dataset demonstrate that our method significantly outperforms existing lightweight networks, achieving a $\delta_{1}$ accuracy of 0.961 with only 2.8 M parameters. The reparameterization strategy in RepAC enables efficient training-time feature learning through four parallel convolution branches while maintaining deployment efficiency through structural reparameterization into a single convolution. The integration of SECDC blocks with optimized dilation patterns further enhances the model’s ability to perceive multi-scale objects and handle challenging scenarios such as moving objects in close proximity to the camera. Our purely convolutional approach demonstrates that effective depth estimation can be achieved without complex attention mechanisms, offering excellent computational efficiency for resource-constrained environments. RepACNet achieves a trade-off between model complexity, inference speed, and depth estimation accuracy, making it particularly suitable for real-time applications. The proposed pure CNN architecture opens new avenues for designing parameter-efficient depth estimation models that maintain simplicity while delivering superior performance.

## Figures and Tables

**Figure 2:**
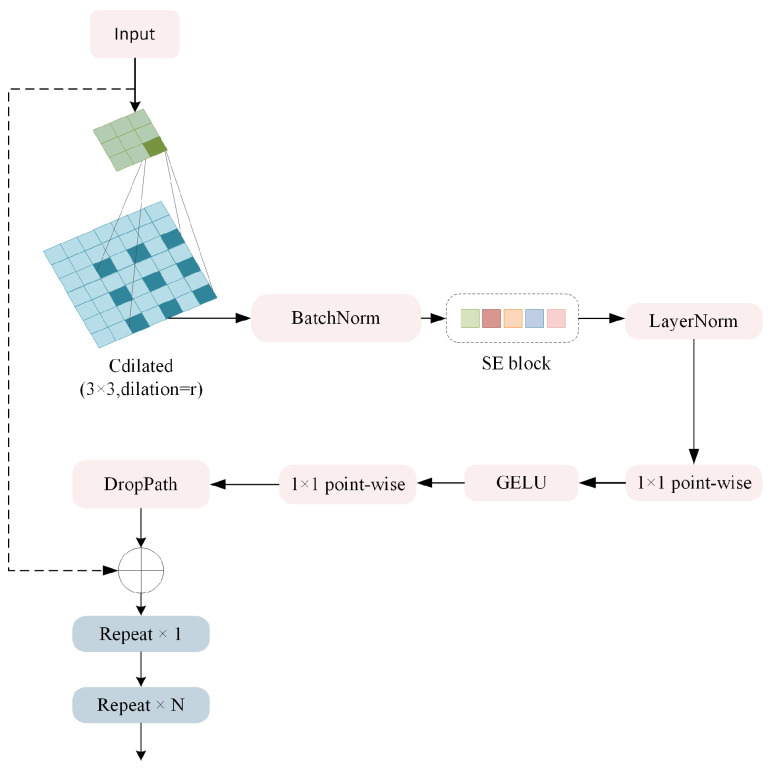
Structures of the proposed Squeeze-and-Excitation Consecutive Dilated Convolution (SECDC) module. In each stage the SECDC module with a different dilation rate is repeated for *N* times.

**Figure 3:**
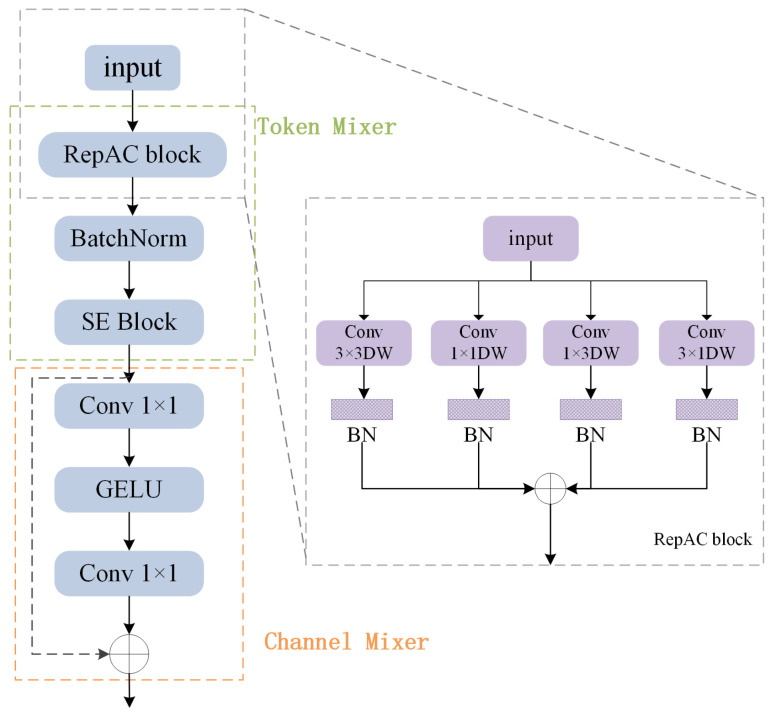
Structures of the Reparameterized Token Mixer with Asymmetric Convolutions (RepTMAC) module. It is divided into two parts: Token Mixer and Channel Mixer, with the RepAC module included in the Token Mixer.

**Table 1:** Stages of the proposed RepACNet. [3 × 3,C] × N means that a SECDC block uses the 3 × 3 kernel size to output C channels, and repeats for N times. There listed the dilation rate of each SECDC block.

Stages	Layers	Configuration	Size
Stage1	Conv Stem	stride = 2	H/2×W/2×48
		[3×3,48]×2	
Stage2	Downsampling	stride = 2	H/4×W/4×48
	SECDC block ×3	[3×3,48]×3	
	RepTMAC block	dilation = 1, 2, 3	
Stage3	Downsampling	stride = 2	H/8×W/8×80
	SECDC block ×3	[3×3,80]×3	
	RepTMAC block	dilation = 1, 2, 3	
Stage4	Downsampling	stride = 2	H/16×W/16×128
	SECDC block ×9	[3×3,128]×9	
	RepTMAC block	dilation = 1, 2, 3, 1, 2, 3, 2, 4, 6	

**Table 4:** Ablation study on model architectures. All the models are trained and tested on NYU Depth v2 with the input size 640 × 192. The symbols “↑/↓” mean that a higher/lower value is better.

Architecture	Abs Rel ↓	Sq Rel ↓	RMSE ↓	$\delta_{1}$ ↑	$\delta_{2}$ ↑	$\delta_{3}$ ↑	Params ↓	GFLOPs ↓
RepACNet full model	**0.123**	**0.075**	**0.423**	**0.852**	**0.974**	**0.995**	2.812	5.623
w/o RepAC	0.148	0.106	0.519	0.778	0.950	0.990	2.809	5.618
w/o SECDC	0.131	0.083	0.456	0.826	0.968	0.994	2.788	5.578
w/o skip connections	0.126	0.079	0.444	0.841	0.971	0.995	**2.748**	**5.497**
w/o RepTMAC	0.148	0.106	0.505	0.791	0.954	0.989	2.995	5.991

## Data Availability

The data presented in this study are available upon request from the corresponding author.

## Abbreviations

The following abbreviations are used in this manuscript:

MDE	Monocular Depth Estimation
CNN	Convolutional Neural Network
RepACNet	Reparameterized Asymmetric Convolution Network
RepTMAC	Reparameterized Token Mixer with Asymmetric Convolution
SECDCs	Squeeze-and-Excitation Consecutive Dilated Convolutions
SE	Squeeze-and-Excitation
BN	Batch Normalization
SILog	Scale-Invariant Logarithmic
RMSE	Root Mean Square Error
AbsRel	Absolute Relative Error
SqRel	Squared Relative Error
